# Prevalence and clinicopathological characteristics of breast cancer patients with brain metastases in Ghana: A single-center cross-sectional study

**DOI:** 10.1371/journal.pone.0329308

**Published:** 2025-08-08

**Authors:** Joseph Daniels, Kofi Adesi Kyei, Ronald Walubo, Andrew Yaw Nyantakyi, Edwina Ayaaba Ayabilah, Judith Naa Odey Tackie

**Affiliations:** 1 National Centre for Radiotherapy, Oncology, and Nuclear Medicine, Korle Bu Teaching Hospital, Accra, Ghana; 2 Department of Radiography, University of Ghana, Accra, Ghana; 3 Department of Oncology, Cape Coast Teaching Hospital, Cape Coast, Ghana; Teesside University, CHINA

## Abstract

Breast cancer remains a leading cause of cancer-related mortality among women globally. While advances in early diagnosis and systemic therapies have improved survival, they have also increased the likelihood of brain metastases over time, particularly in low-resource settings where limited diagnostic and treatment capacity exacerbates the burden of late-stage disease. The study aimed to determine the prevalence and describe the clinicopathological characteristics of breast cancer patients diagnosed with brain metastasis in a limited-resource healthcare setting. This research was a single-institution-based quantitative cross-sectional study. Socio-demographic, clinical and pathological data were extracted from patients’ medical records as well as the hospital-based cancer registry. Data were analyzed using STATA software (version 16). Descriptive and logistic regression analyses were performed. The study involved 144 adult female metastatic breast cancer patients with a mean age of 48.7 years (SD 11.3). The prevalence of brain metastasis was 17.5%. Only 4.9% presented with de novo brain metastasis, p < 0.001. Bone metastases were present in 31.9% whereas 26.4% and 12.5% had concurrent lung and liver metastasis respectively. In all, 38.9% had grade III tumors. Also, 50.6% were categorized as recursion partition analysis (RPA) class II whereas 49.3% had a performance status of ECOG 2. A considerable majority (86.8%) were treated with palliative intent whereas 13.2% received best supportive care only. In total, 86.8% underwent radiotherapy whereas 81.3% received systemic treatments, with chemotherapy being the most frequently utilized modality (73.5%). Most patients (88%) were treated with 2-dimensional radiotherapy whereas 3.2% received hippocampal-sparing intensity-modulated radiotherapy. The high prevalence of brain metastasis among breast cancer patients with distant metastases reflects the challenges associated with late-stage breast cancer presentation and limited access to advanced diagnostic and therapeutic interventions in limited-resource healthcare settings.

## Introduction

Breast cancer is the most frequently diagnosed cancer globally, with an estimated 2.3 million new cases diagnosed annually [1]. Bone and visceral organs as well as the brain are the commonest sites of breast cancer metastasis [2]. Notably, breast cancer is the second most frequent cause of brain metastases, after lung cancer [3]. The risk of developing brain metastasis ranges from 10 to 16% among patients with advanced breast cancer [4]. Risk factors of breast cancer patients for developing brain metastases include young age at diagnosis, multiple extracranial metastatic sites, high tumor grade, large tumor size, nodal involvement, multiple primary sites, and specific molecular subtypes such as human epidermal growth factor receptor 2 – positive (HER-2/neu+) and triple negative breast cancers (TNBC) [5]. Routine screening for brain metastases in breast cancer patients is generally not recommended due to the absence of strong evidence linking early detection with improved overall survival outcomes. Matsumoto et al. (2008) demonstrated that the detection of asymptomatic brain metastases did not lead to a meaningful extension of survival in breast cancer patients, as brain metastases are often aggressive and challenging to treat [6]. Similarly, Miller et al. (2003) found that even with advanced imaging techniques, early detection rarely altered the overall course of treatment in a way that significantly prolonged life expectancy [7]. Niikura et al. (2014) also supported this conclusion, noting that most therapies available for brain metastases are generally palliative and do not drastically alter the trajectory of the disease [8]. Moreover, unnecessary screening can expose patients to additional stress, medical risks, and healthcare costs without any clear benefits. Therefore, in clinical practice, screening for brain metastases is usually considered only when neurological symptoms arise, or in patients with subtypes like HER-2/neu+ or TNBC, which are known to have a higher risk of brain metastasis [9,10].

Treatment options for brain metastases include surgery, radiotherapy, chemotherapy, immunotherapy, and targeted as well as hormonal therapy [11]. While whole brain radiotherapy (WBRT) can provide symptomatic relief and modest control of intracranial disease, newer approaches such as stereotactic radiosurgery (SRS) are increasingly preferred where available, especially for patients with a limited number of brain metastases. However, for patients with large or symptomatic lesions, surgical resection offers better local control compared to radiotherapy alone. Guidelines from the American Society of Clinical Oncology (ASCO) and the Society for Neuro-Oncology (SNO) recommend surgical intervention followed by focused radiation to achieve optimal outcomes [11,12]. Systemic therapies including chemotherapy, hormonal therapy, and targeted agents, have shown limited efficacy in treating brain metastases due to the blood-brain barrier, which restricts the penetration of these drugs [13]. Given the limited survival benefit of existing treatments, the focus in managing brain metastases often shifts toward preserving quality of life and neurological function [14]. The combination of SRS and systemic therapies is emerging as a preferred strategy, particularly in patients with good performance status and limited extracranial disease [15]. Unfortunately, for many breast cancer patients with brain metastasis, the prognosis remains poor, with estimated survival less than one year, even with appropriate therapy [16]. Late-stage presentation is common in Ghana, attributable to limited awareness, socio-cultural beliefs, and restricted access to specialized care. Geographic disparities and financial constraints also impede timely diagnosis and treatment, exacerbating disease progression and mortality. Additionally, there is limited access to screening services in Ghana, as well as sociocultural barriers that delay timely care-seeking. These context-specific factors make a study of brain metastases in Ghanaian breast cancer patients particularly important, as the burden and progression patterns may differ from those observed elsewhere.

Brain metastasis significantly impacts patients’ quality of life, causing symptoms like seizures, paralysis, and cognitive impairment, posing a substantial burden on healthcare resources and caregivers. The challenges associated with managing these symptoms as well as the role of multidisciplinary care and the importance of palliative services for patients with brain metastasis cannot be overemphasized [17]. However, in Ghana, there is a paucity of reliable data on the prevalence as well as clinical and pathological profiles of patients with metastatic breast cancer to the brain. The aim of the study was to determine the prevalence and describe the socio-demographic as well as clinico-pathological characteristics of patients with breast cancer who were diagnosed with brain metastasis at a large radiotherapy and oncology centre in sub-Saharan Africa, thereby addressing a critical gap in the literature and informing local healthcare strategies.

## Methods

### Study design and setting

The research was a single-institution-based quantitative cross-sectional study conducted at a large oncology and radiotherapy centre in sub-Saharan Africa. The centre operates a 6 MV linear accelerator and a 1.25 MeV cobalt-60 teletherapy machine that are used in the delivery of radiotherapy for brain metastases. Brain radiotherapy techniques employed comprise 2-dimensional WBRT with two lateral opposing fields as well as 3-dimensional conformal and hippocampal-sparing intensity-modulated radiotherapy (HS-IMRT). Access to comprehensive cancer care remains a significant challenge in this setting due to limitations in infrastructure, personnel, and treatment resources. Diagnostic services are primarily reliant on immunohistochemistry, with molecular testing rarely available due to high costs and limited facilities. Available treatment options for brain metastasis include various chemotherapeutic agents, endocrine therapy (mainly Tamoxifen, Anastrozole and Exemestane), and radiotherapy (2D, 3D-CRT and IMRT). Modern techniques such as SRS and stereotactic ablative radiotherapy (SABR) are not locally available. The availability and accessibility of targeted therapies and immunotherapeutic agents are constrained by seemingly insurmountable economic barriers.

### Study population

The study included only adult female patients (≥ 18 years) with a histologically confirmed diagnosis of breast cancer and radiologically confirmed brain metastases managed over a five-year period (January 2017 – December 2021) at the National Centre for Radiotherapy, Oncology and Nuclear Medicine, Ghana. Eligible patients were identified through a comprehensive review of the institutional cancer registry and medical records database. Patients diagnosed with breast cancer without evidence of metastatic spread to the brain were excluded, as were those with brain metastases originating from a non-breast primary malignancy or an unknown primary site. Additionally, patients diagnosed with primary brain tumors were also excluded from the study. Patients with incomplete or unavailable clinical and pathological records, including imaging studies necessary to confirm the diagnosis of brain metastases, were also excluded.

### Sampling technique and study size

**Fig 1:**
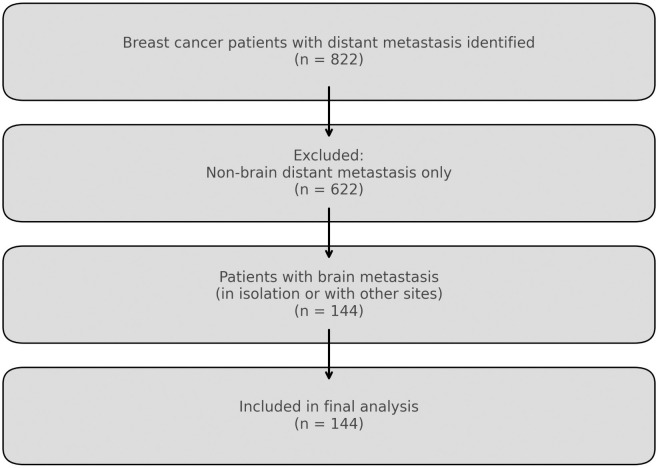
Diagram illustrating the selection of breast cancer patients with brain metastases for inclusion in the cross-sectional study.

Overall, there were 822 breast cancer patients with confirmed distant metastasis. A total population sampling technique was used to recruit all 144 eligible breast cancer patients with confirmed brain metastasis (Fig 1). This approach was chosen to ensure comprehensive coverage, as every patient diagnosed with brain metastases secondary to breast cancer, and meeting the inclusion criteria, was included.

### Bias

Total population sampling technique, which was used to purposively recruit all patients in the eligible population reduced potential selection bias. Screening of patients’ records for eligibility and data collection were conducted by two independent reviewers to confirm adherence to the predefined inclusion and exclusion criteria. Additionally, a subset (30%) of the data collected was re-verified by a third reviewer to ensure accuracy and consistency, thus minimizing the risk of data entry errors. All eligible patients managed within the study period, regardless of disease severity or treatment status, were considered for inclusion. Patients were excluded only if critical data were unavailable despite extensive retrieval attempts from institutional archives. This rigorous selection and data collection process provided a transparent and comprehensive characterization of the study population, further reducing potential selection bias and ensuring robust analysis of the patient population.

### Variables

The prevalence of brain metastasis was defined as the ratio of breast cancer patients diagnosed with brain metastasis to the total number of breast cancer patients with distant metastasis, i.e., $\frac{Number\;of\;breast\;cancer\;patients\;with\;brain\;metastasis}{Total\;number\;of\;breast\;cancer\;patients\;with\;distant\;metastasis}\times 100\;\%$. Performance status was assessed using the standardized Eastern Cooperative Oncology Group (ECOG) scale [18] to evaluate patients’ overall health and ability to perform daily activities. The recursive partitioning analysis (RPA) scoring system was used to classify patients into three prognostic classes that are associated with distinct median survival outcomes [19]. Class I included patients who were < 65 years old, with a Karnofsky performance status (KPS) ≥70%, and no evidence of extracranial disease – associated with the best prognosis, with a median survival of approximately 12 months. Class II comprised patients who were either ≥ 65 years or had a KPS score < 70%, with or without active extracranial disease – associated with a median survival of about 6 months. Finally, RPA Class III consisted of patients with poor prognostic indicators, such as a KPS < 70%, the presence of active extracranial disease, or those > 65 years with significant functional impairment – associated with the worst prognosis and a median survival of approximately 3 months.

### Data collection

The study utilized the data of patients diagnosed with metastatic breast cancer to the brain between 2017 and 2021, extracted from hospital-based medical records on 29^th^ July, 2023. Data collection involved detailed examination and validation of histopathological reports, imaging studies, and clinical records. Information regarding the total number of breast cancer patients diagnosed with distant metastasis was obtained from the hospital-based cancer registry. Data systematically recorded for each patient included demographic information (age at diagnosis, ethnicity, religion, insurance status, and employment status), histopathological data (histological diagnosis and tumor grade), molecular subtype, metastatic profile (sites of extracranial metastasis), and treatment received after the diagnosis of brain metastasis.

### Data management and analysis

Data management procedures included the meticulous review of patients’ records to ensure the completeness and accuracy of extracted data. Patients with incomplete or missing clinical and/or pathological records were excluded from the analysis to maintain the integrity of the dataset. All data were securely stored to protect patient confidentiality. Data were anonymized, coded, statistically cleaned, and analyzed using STATA version 16 for Microsoft Windows (College Station, TX: Stata Corp LLC), following best practices for epidemiologic analysis [20].

Descriptive statistics, including mean and standard deviation were calculated for continuous variables, while frequency and percentages were determined for categorical variables. Multivariable logistic regression was performed to evaluate the association between patient clinicopathological characteristics and the presence of brain metastases (binary outcome: yes/no). Predictor variables included age group, histological grade, histological subtype, molecular subtype (Luminal A/B, HER-2-enriched, and TNBC), and the presence of bone, lung, and liver metastases. Backward stepwise regression was applied to identify significant predictors, with adjusted odds ratios (ORs), 95% confidence intervals (CIs), and p-values reported for each predictor. Statistical significance was determined at *p* < 0.05.

### Ethical considerations

Ethical approval was obtained from the institutional review board of the School of Biomedical and Allied Health Sciences, University of Ghana, Legon. Written informed consent was obtained directly from patients or their legally authorized representatives, in instances where patients were deceased. The research adhered to the principles outlined in the Declaration of Helsinki [21], ensuring that the rights and welfare of participants were prioritized.. All data were fully anonymized to remove all traces of patient-identifying information prior to analysis to protect patients’ privacy and the confidentiality of their medical records.

## Results

### Socio-demographic characteristics and prevalence of brain metastasis

The study involved 144 female breast cancer patients with confirmed metastasis to the brain (+/- other secondary sites). The mean age was 48.7 years (SD 11.3). A considerable majority were between 50 and 59 years (31.9%) as summarized in Table 1. The vast majority were Ghanaians (83.3%). Regarding ethnicity, 36.1% identified as Akan, 26.4% as Ga, and 13.9% as Ewe. The predominant religion was Christianity, practiced by 84%, whereas 16% identified as Muslim. In all, 72% were employed, whereas 28% were unemployed. A considerable majority had health insurance coverage (74%). The prevalence of brain metastasis among breast cancer patients with distant metastasis was 17.5%.

**Table 1:** Socio-demographic characteristics (N = 144).

Characteristics	Variables	Frequency (n)	Percentage (%)
**Age (Years)**	< 40	36	25
	40–49	36	25
	50–59	46	31.9
	60–69	20	13.9
	≥ 70	6	4.2
**Ethnicity**	Akan	52	36.1
	Ga	38	26.4
	Ewe	20	13.9
	*Other	10	6.9
	Non-Ghanaian	24	16.7
**Religious affiliation**	Christian	121	84.0
	Muslim	23	16.0
**Employment status**	Unemployed	40	28.0
	Employed	104	72.0
**Health Insurance**	Insured	107	74
	Uninsured	37	26

Akan, Ga, and Ewe are distinct ethnic groups in Ghana. *“Other” includes additional minority ethnic groups not individually specified.

### Clinical characteristics

Table 2 illustrates the distribution of metastatic sites, tumor grade, histological type, and molecular subtype within the study population, highlighting the clinical diversity of breast cancer patients with brain metastases. A considerable majority presented with progressive disease following prior breast cancer treatment (93.1%) whereas only 4.9% presented with de novo brain metastasis (4.9%). Concurrent bone metastases were present in 31.9% of the patients whereas 26.4% and 12.5% had simultaneous lung and liver metastasis respectively. A significant proportion had grade III tumors (38.9%). The most prevalent histological type was invasive carcinoma of no special type (NST), accounting for 76.4%, whereas 8.3% had invasive lobular carcinoma (ILC).

**Table 2:** Clinical characteristics of the study participants (N = 144).

Characteristics	Variables	Frequency (n)	Percentage (%)
**Metastatic setting**	De novo metastasis	7	4.9
	Progressive disease	134	93.1
**Bone Metastasis**	Absent	98	68.1
	Present	46	31.9
**Lung Metastasis**	Absent	106	73.6
	Present	38	26.4
**Liver Metastasis**	Absent	126	87.5
	Present	18	12.5
**Histological grade**	I	10	6.9
	II	50	34.7
	III	56	38.9
	Not specified	28	19.4
**Histology**	Invasive** **carcinoma, NST	110	76.4
	Invasive lobular** **carcinoma	12	8.3
	* Other histological types	22	15.3
**Molecular subtype**	Luminal A/B	47	32.6
	HER-2/neu - enriched	63	43.7
	Triple negative	34	23.61

NST: no special type, HER-2/neu: human epidermal growth factor receptor 2. *Other histological types included metaplastic, mixed, mucinous, and cribriform carcinoma. Luminal A – characterized by hormone receptor positivity (estrogen and/or progesterone receptor-positive), HER-2/neu negativity, and Ki 67 ≤ 14%. Luminal B-like – characterized by either [hormone receptor positivity, HER-2/neu negativity, and high Ki 67 (>14%)] or [hormone receptor positivity, HER-2/neu positivity and any Ki 67].

**Fig 2:**
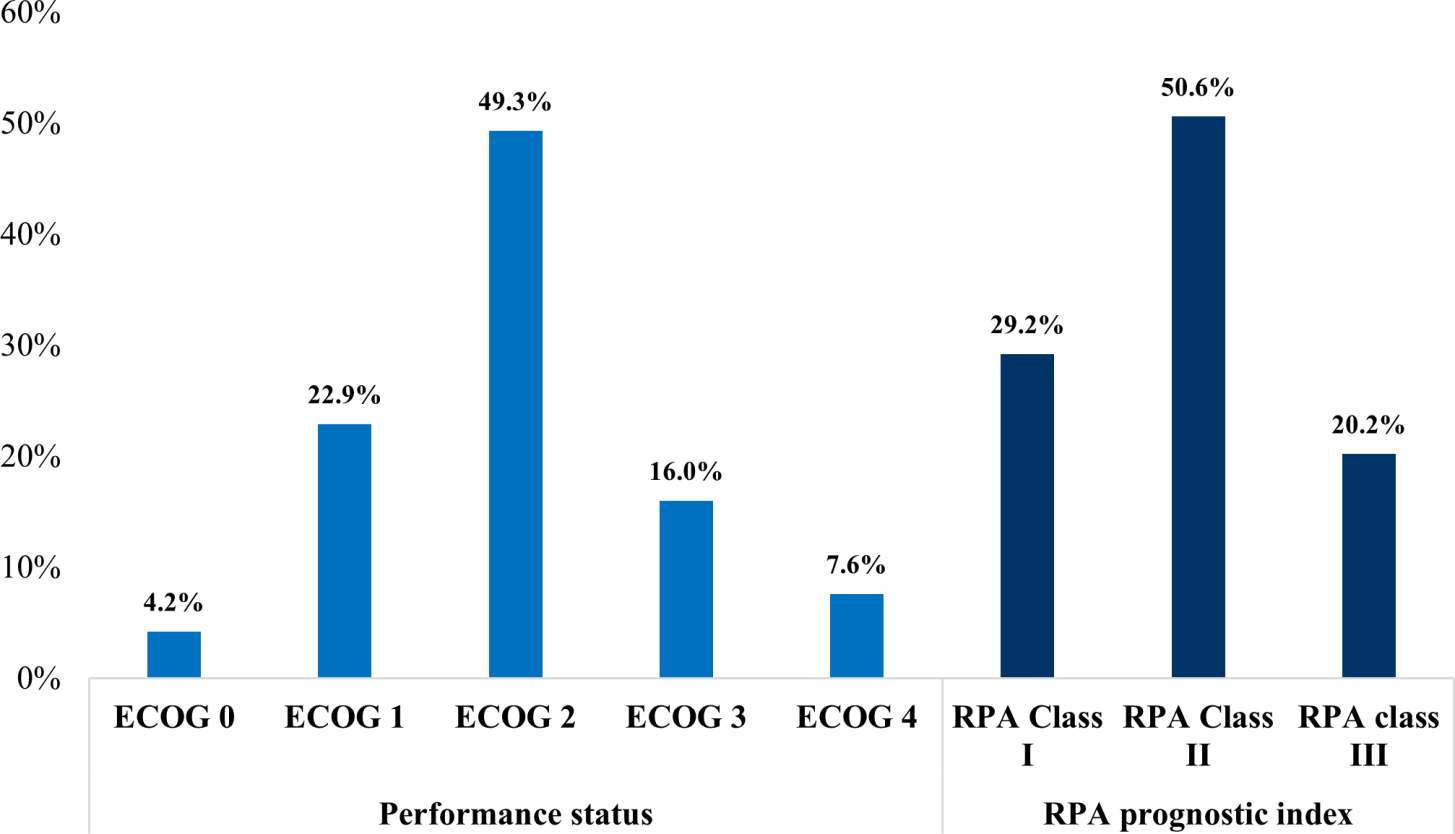
Performance status and prognostic index of the study participants (N = 144). ECOG: Eastern Cooperative Oncology Group, RPA: recursion partition analysis.

Fig 1 highlights two important clinical characteristics of breast cancer patients with brain metastases: ECOG performance status and RPA prognostic index [22]. Most patients were ECOG 2 (49.3%), indicating moderate limitations in daily activities. A smaller proportion were ECOG 3 (16.0%) reflecting more severe physical debilitation, being bedridden or unable to perform self-care. In all, 50.6% were classified as RPA Class II, suggestive of intermediate survival prospects whereas 20.2% were categorized as RPA Class III, representing the group with the poorest prognosis (Fig 2).

### Treatment-related characteristics

A considerable majority (86.8%) received treatment with palliative intent, whereas 13.2% received best supportive care alone (Table 3). None of the patients was treated with curative intent, neither were any treated with surgical interventions. In all, 86.8% underwent palliative radiotherapy whereas 81.3% received palliative systemic therapy. Most patients (88%) were treated with 2D-WBRT, whereas 8.8% received 3D-WBRT and 3.2%, HS-IMRT. A considerable majority (88%) were treated with the cobalt-60 teletherapy machine, whereas 12% received treatment with the 6 MV linear accelerator. Among patients receiving palliative systemic therapy, chemotherapy was the most frequently used modality (73.5%), followed by targeted (45.3%), hormonal (30.8%), and immunotherapy (6.0%).

**Table 3:** Treatment-related characteristics (N = 144).

Characteristics	Variables	Frequency (n)	Percentage (%)
Treatment intent	Curative	0	–
	Palliative	125	86.8
	Best supportive care alone	19	13.2
*Treatment modality for palliative therapy	Surgery	0	–
	Radiotherapy	125	86.8
	Systemic therapy	117	81.3
	No cancer-specific treatment	19	13.2
RT technique (N = 125)	2D WBRT	110	88.0
	3D WBRT	11	8.8
	HS-IMRT	4	3.2
	SABR	0	–
	SRS	0	–
RT machine (N = 125)	Cobalt-60	110	88
	6 MV linear accelerator	15	12
Palliative systemic therapies (N = 117)	Targeted therapy	53	45.3
	Chemotherapy	86	73.5
	Immunotherapy	7	6.0
	Hormonal therapy	36	30.8

*The treatment modalities and systemic therapies used after the diagnosis of brain metastasis were not mutually exclusive. Patients were treated with radiotherapy and/or systemic therapy. Similarly, patients receiving systemic therapy were treated with targeted therapy and/or chemotherapy and/or immunotherapy and/or hormonal therapy. 2D: 2-dimensional, 3D: 3-dimensional, WBRT: whole brain radiotherapy, HS-IMRT: hippocampal-sparing intensity-modulated radiotherapy, SABR: stereotactic ablative radiotherapy, SRS: stereotactic radiosurgery.

### Multivariable analysis of factors associated with brain metastases

The multivariable logistic regression model showed that patients aged 40–49 and 50–59 years had significantly lower odds of presenting with brain metastases compared to those under 40 years, with adjusted ORs of 0.08 (95% CI: 0.02–0.36, p = 0.002) and 0.06 (95% CI: 0.02–0.27, p = 0.003), respectively (Table 1). Notably, TNBC was independently associated with higher odds of brain metastasis when compared to Luminal A/B subtypes (adjusted OR: 3.12, 95% CI: 1.05–13.05, p = 0.001) (Table 4).

**Table 4:** Multivariable analysis of clinicopathological characteristics (N = 144).

Characteristics	Crude			Adjusted		
	OR	95% CI	*p* – value	OR	95% CI	*p* -value
**Age (Years)**						
<40	*Ref*			*Ref*		
40–49	0.06	[0.01–0.32]	0.001	0.08	[0.02–0.36]	0.002
50–59	0.05	[0.01–0.24]	0.001	0.06	[0.02–0.27]	0.003
60–69	0.12	[0.03–0.62]	0.011	0.14	[0.04–0.72]	0.028
≥ 70	0.56	[0.09–3.45]	0.531	0.57	[0.11–3.48]	0.512
**Bone Metastasis**						
No	*Ref*			*Ref*		
Yes	1.03	0.39 to 2.53	0.198	1.08	0.37 to 2.51	0.197
**Lung Metastasis**						
No	*Ref*			*Ref*		
Yes	2.16	0.87 to 5.36	0.094	2.11	0.85 to 5.31	0.083
**Liver Metastasis**						
No	*Ref*			*Ref*		
Yes	0.56	0.12 to 2.60	0.200	0.54	0.14 to 2.62	0.221
**Pathology Type**						
Ductal	*Ref*			*Ref*		
Lobular	2.54	0.72 to 8.96	0.147	2.52	0.71 to 8.92	0.145
Other	0.14	0.03 to 0.67	0.013	0.15	0.04 to 1.01	0.067
**Molecular Subtype**						
Luminal A/B	*Ref*			*Ref*		
HER-2-enriched	0.34	0.08 to 1.44	0.144	0.32	0.07 to 1.41	0.146
Triple Negative	3.07	1.04 to 14.06	0.001	3.12	1.05 to 13.05	0.001

*OR: odds Ratio, CI: confidence interval, HER-2: human epidermal growth factor receptor 2. The crude & adjusted odds ratios (OR) and 95% confidence intervals (CI) reflect the relative likelihood of specific characteristics being associated with brain metastases within this patient group, rather than comparisons with patients who do not have brain metastases.*

## Discussion

There was a 17.5% prevalence of brain metastasis among breast cancer patients with metastatic disease, consistent with the 10–30% range reported in other studies [23]. In low-resource settings, breast cancer patients experience higher prevalence of brain metastasis, partly due to later-stage diagnosis, lack of early screening programs and limited access to advanced diagnostic tools, such as MRI, and advanced treatments like SABR & SRS [24,25]. Late presentation, limited healthcare access, socioeconomic barriers, and suboptimal treatment options also contribute to the prevalence in limited-resource settings. However, in high-income countries, more widespread use of adjuvant and neoadjuvant therapies have reduced the occurrence of brain metastasis, even though it still remains a significant concern for high-risk groups, such as those with TNBC [26,27]. The study demonstrated a younger mean age of 48.7 years (SD 11.3), suggesting that aggressive disease affects premenopausal women disproportionately. Premenopausal patients are more likely to have hormone receptor-negative and HER-2/neu+ breast cancers, which are associated with higher rates of brain metastases. I n contrast, postmenopausal patients typically have hormone receptor-positive tumors, which are less aggressive but still carry a risk of late brain metastases if untreated. A Nigerian study, reported a comparable mean age for metastatic breast cancer patients, reflecting the earlier onset of aggressive disease in African populations compared to high-income countries where the median age for breast cancer brain metastases is usually between 55 and 65 years [28,29]. This age discrepancy may be attributed to factors such as genetic predisposition and disparities in access to timely healthcare in low-resource settings. Additionally, the low proportion of patients between 60 and 69 years suggests that even though older patients are typically at greater risk for metastases overall, disease progression to the brain may be limited by competing mortality from other comorbidities [24]. The underrepresentation of patients ≥70 years (4.2%) aligns with prior research showing that elderly patients tend to receive less aggressive treatment, such as fewer brain-directed interventions (e.g., MRI surveillance), which could contribute to underdiagnosis of brain metastases in this group [30].

In this study, 28% of the patients were unemployed. In an institution-based cross-sectional study of breast cancer patients seen at an oncology centre of North East Ethiopia, it was observed that being unemployed was an important predictor of delayed presentation. Employed breast cancer patients were found to be 0.14 times less likely to delay seeking medical attention or initiating treatment compared to compared to unemployed patients (adjusted OR = 0.19, 95% CI = 0.03–0.91) [31]. In limited-resource settings, unemployment often correlates with reduced access to early cancer screening and treatment [32], whereas in high-income countries, universal healthcare systems can mitigate these disparities by reducing the economic burden of cancer treatment [33].

The high proportion of grade III tumors (38.9%) reflects the aggressive nature of breast cancer in low-resource settings [29]. In comparison, high-income countries report fewer cases of high-grade tumors, likely due to earlier detection and the availability of routine screening [34]. Also, HER-2/neu+ and TNBC subtypes exhibited high brain metastasis propensity, which aligns with known patterns of metastatic behavior. Triple negative breast cancer is widely recognized for its aggressive behavior and poor prognosis, particularly in African populations [26,35]. In low-resource settings, accurate molecular subtype characterization is often limited due to constrained laboratory resources, which might result in delays in the use of targeted therapies that could reduce the risk of brain metastasis [36]. The strong association between TNBC and brain metastasis emphasizes the need for improved management strategies in low-resource settings, especially for this high-risk group. The introduction of HER-2-targeted therapies, such as trastuzumab, have led to improved outcomes for HER-2/neu+ patients, but these therapies may not be widely available in low-resource settings, thereby, contributing to a higher incidence of brain metastasis [37].

In all, 86.8% received treatment with palliative intent, while 13.2% received only best supportive care. This finding is consistent with reports from other low-income countries, where limited access to curative treatments often results in palliative care being the primary management option for metastatic breast cancer [38]. In high-income settings, metastatic patients with low disease-burden are more likely to receive aggressive multimodal treatments, including surgery, SRS, SABR and novel systemic therapies such as targeted and immunotherapy, resulting in improved survival outcomes [27,39,40]. Palliative systemic therapy was predominantly chemotherapy (73.5%), with a smaller proportion of patients receiving targeted therapy (45.3%) and hormonal therapy (30.8%). These findings reflect the limited availability of novel therapies in low-resource settings, where advanced treatments such as immunotherapy (used in only 6% of cases) remain inaccessible to most patients.

The high proportion of patients treated with 2D-WBRT (88%) highlights the reliance on simpler, less precise radiotherapy techniques that are common in resource-constrained settings. In contrast, studies from high-income countries report widespread use of SRS, SABR and HS-IMRT, leading to better control of brain metastases and reduced cognitive side effects [41]. Recent trials have demonstrated the potential benefits of HS-IMRT in preserving neurocognitive function without compromising tumor control, though its availability remains a challenge outside high-income countries [42]. Additionally, SRS and SABR, which are increasingly adopted to manage limited brain metastases with minimal neurotoxicity [43], are largely inaccessible in many low-resource environments due to financial and infrastructure barriers [44]. The heavy reliance on cobalt-60 teletherapy (88%) reflects a common trend in low-resource environments, as linear accelerators (used by only 12% of the participants) are often scarce due to high acquisition and maintenance costs. Although cobalt-60 machines are functional, they lack the precision of modern linear accelerators, potentially leading to higher radiation exposure to healthy tissues and increased side effects, which could contribute to poorer patient outcomes.

The results of the study highlight critical disparities in the management of breast cancer patients with brain metastases between low- and high-income settings. Limited access to advanced diagnostic and therapeutic modalities significantly hamper the management of metastatic breast cancer in sub-Saharan Africa [36]. Advanced imaging techniques, such as contrast-enhanced MRI, are essential for the early detection of brain metastases. However, the cost and accessibility of such technologies often limit their use in resource-constrained environments. Consequently, many patients may only receive a diagnosis of brain metastasis once neurological symptoms become pronounced, at which point the disease may be at an advanced stage with limited therapeutic options being available [45]. More effort must be directed toward improving early detection, enhancing access to novel & effective systemic therapies, and expanding radiotherapy infrastructure. Partnerships between high- and low-income countries, alongside investment in local healthcare infrastructure, are essential to bridge the gap in cancer outcomes [25]. Technology transfer initiatives could also bridge these gaps over time, enabling more patients to benefit from state-of-the-art cancer care. Implementing tailored treatment strategies based on molecular subtypes, particularly for high-risk groups such as TNBC and HER-2/neu+ patients, may also help mitigate the burden of brain metastases in low-income countries. Early and aggressive management of breast cancer itself, including the use of chemotherapy, endocrine, and HER-2-targeted therapies, can reduce the risk of distant metastasis, including brain metastasis. The implementation of such treatments in low-resource healthcare settings is often inconsistent, leading to higher rates of advanced disease [46]. This further reinforces the importance of improving cancer care infrastructure and fostering international collaborations to provide low-cost or subsidized therapies for metastatic breast cancer in resource-constrained settings.

### Limitations

The study was conducted at a single institution in a low-resource setting, which may limit the generalizability of the findings to other regions. Access to neuroimaging may have been limited, and some breast cancer patients with brain metastasis at the study site may have gone undiagnosed, potentially underestimating the true prevalence of brain metastasis among breast cancer patients. Additionally, the population described may reflect patients with greater access to specialized oncology care, potentially excluding underserved populations or those in rural areas.

### Recommendations

Efforts to improve early diagnosis, provide targeted therapies, and enhance access to neuroimaging are essential to reducing the burden of brain metastasis in low-resource environments. Establishing a national breast cancer-specific registry that includes standardized clinical, pathological, molecular, and imaging data would enhance the understanding of metastatic patterns, including brain involvement. Given the limitations of this single-center, retrospective study, future research should aim to conduct prospective, multicenter investigations across Ghana and the broader West African region to validate these findings and improve generalizability. Future studies should also focus on the growing need for affordable and accessible interventions in low-resource settings to improve outcomes for metastatic breast cancer patients, including enhanced diagnostic capabilities and equitable access to systemic therapies targeting specific cancer subtypes, particularly HER-2/neu+ and hormone receptor-positive breast cancers. Additionally, the survival outcomes of breast cancer patients diagnosed with brain metastasis in sub-Saharan Africa should be comprehensively evaluated. Future research may also explore a comparative analysis to elucidate differences between patients diagnosed with breast cancer with and without brain metastases in Ghana.

## Conclusions

The study underscores the complex interplay of socio-demographic and clinical factors in the occurrence of brain metastases in breast cancer patients in a low-resource setting. Comparative analyses with other studies highlight the urgent need for improved healthcare access and advanced treatments to address the disparities in breast cancer management between low- and high-income countries. The prevalence of brain metastasis among breast cancer patients with distant metastasis was 17.5%, reflecting the challenges associated with late-stage breast cancer presentation and the limited access to advanced diagnostic and therapeutic interventions in low-resource settings. As precision therapies evolve, this study highlights the critical need for improved access to advanced radiotherapy equipment and systemic agents, which could enhance the prognosis of patients with brain metastases.
